# Antioxidant, anti-inflammatory and immunomodulatory effects of spirulina in exercise and sport: A systematic review

**DOI:** 10.3389/fnut.2022.1048258

**Published:** 2022-12-14

**Authors:** Patrizia Calella, Giuseppe Cerullo, Mirella Di Dio, Fabrizio Liguori, Valeria Di Onofrio, Francesca Gallè, Giorgio Liguori

**Affiliations:** ^1^Department of Movement Sciences and Wellbeing, University of Naples Parthenope, Naples, Italy; ^2^Department of Economics and Legal Studies, University of Naples Parthenope, Naples, Italy; ^3^Department of Sciences and Technologies, University of Naples Parthenope, Naples, Italy

**Keywords:** spirulina, antioxidant, immunomodulatory, anti-inflammatory, athletes

## Abstract

*Arthrospira platensis*, also known as spirulina, is currently one of the most well-known algae supplements, mainly due to its high content of bioactive compounds that may promote human health. Some authors have hypothesized that spirulina consumption could protect subjects from exercise-induced oxidative stress, accelerate recovery by reducing muscle damage, and stimulate the immune system. Based on this, the main goal of this review was to critically analyze the effects of spirulina on oxidative stress, immune system, inflammation and performance in athletes and people undergoing exercise interventions. Of the 981 articles found, 428 studies were considered eligible and 13 met the established criteria and were included in this systematic review. Most recently spirulina supplementation has demonstrated ergogenic potential during submaximal exercise, increasing oxygen uptake and improving exercise tolerance. Nevertheless, spirulina supplementation does not seem to enhance physical performance in power athletes. Considering that data supporting benefits to the immune system from spirulina supplementation is still lacking, overall evidence regarding the benefit of spirulina supplementation in healthy people engaged in physical exercise is scarce and not consistent. Currently, spirulina supplementation might be considered in athletes who do not meet the recommended dietary intake of antioxidants. Further high-quality research is needed to evaluate the effects of spirulina consumption on performance, the immune system and recovery in athletes and active people.

**Systematic review registration:**

[https://www.crd.york.ac.uk/prospero/display_record.php?RecordID=262896], identifier [CRD42021262896].

## Introduction

*Arthrospira platensis*, commonly named spirulina (SP), is a type of blue-green algae, which is a member of the phylum Cyanobacteria (1). It contains proteins (phycocyanin), B-group vitamins, natural colors (chlorophyll and carotenoids), and important fatty acids. Since the 16th century, SP has been generally accepted as a food and dietary supplement (2). It is used as a nutraceutical food supplement due to its high protein (up to 65% dry weight) and bioactive compound content including many phytonutrients, such as β-carotene, echinenone, zeaxanthin, 3-hydroxyechinenone, c-phycocyanin, which all have strong antioxidant activity (3). It has been reported that SP improves macrophage activity, natural-killer (NK) cells proliferation, activation of T-cells, up-regulating key cells and organs of the immune system enhancing their ability to counteract the action of infectious agents and the activity of environmental toxins. The potential therapeutic applications of SP, based on its immunomodulatory and anti-inflammatory activities, have already been reviewed by several authors (4–7).

Beyond the clinical implications, some authors have hypothesized that SP supplementation could also be advantageous for healthy, active individuals, especially athletes. For example, SP could modulate markers of exercise-induced lipid peroxidation, such as plasma thiobarbituric acid reactive substances (TBARS), malondialdehyde (MDA) and protein carbonyls (PC), as well as improving the activity of redox enzymes such as catalase (CAT), glutathione peroxidase (GPx) and superoxide dismutase (SOD), suggesting a role in the management of oxidative stress (8, 9). People involved in high-intensity physical training increase the production of reactive oxygen species (ROS) and need to follow a well-balanced diet that satisfies their requirements for energy, macro- and micronutrients, in order to maintain an optimal redox state and avoid potential immune dysfunction (10). However, bearing in mind that ROS production during exercise has a pivotal role in long-term training adaptation (11–13), excessive oxidative damage could have a negative impact on the immune system (14), recovery (15), ability to perform and general health (15, 16). According to anecdotal evidence, the Chinese and Cuban Olympic teams have been taking SP daily for many years and have performed better (17). Early *in vitro* reports demonstrating the high radical scavenging activity of the algae (18), probably involving the activation of the NRF2 signaling pathway (6, 19) and the prevention of lipid peroxidation (18), have sparked a lot of interest in how the antioxidant effects of SP supplementation may support exercise performance. Other authors have shown that SP could accelerate recovery by reducing creatine kinase (CK), lactate dehydrogenase (LDH) and C-reactive protein (CRP) (9, 20); strengthen the immune system (21–23); and improve performance in various situations (9, 24).

The potential effects of SP supplementation on performance, immunity, exercise-induced muscle damage, and recovery in athletes have not been considered in the recommendations published so far (25–29). Only Braakhaius et al. and Gurney et al. discussed the impact of SP supplementation on athletic performance, focusing on its antioxidant and ergogenic properties (30, 31).

To our knowledge, the effects of SP supplementation in athletes and healthy people engaged in exercise have not been systematically reviewed; thus, the main aim of this review is to critically appraise the literature on the exercise-related effects of SP supplementation on oxidative stress, immune system, inflammation and performance.

## Materials and methods

The Preferred Reporting Items for Systematic Reviews and Meta-Analyses (PRISMA) guidelines were followed for this systematic review (32) also taking into account the updating in the motor-sports field and the 27 points of the PERSiST guidelines (33). The research protocol has been registered in the PROSPERO database (reg. n. CRD42021262896) with limitations.

### Eligibility criteria

For this review studies have been included which investigated the effects of SP supplementation in humans of any age, who regularly played sports at any level or underwent exercise interventions, without diagnosed pathologies or disorders. Only randomized controlled studies reporting the dose of SP administered were included in the analysis.

### Outcomes

How SP affects the modulation of oxidative stress, exertional inflammation, the regulation of the immune system and whether it has effects on performance and fatigue.

### Literature search and selection of studies

The search for literature was carried out until 23 July 2022 on the following electronic databases: (1) MEDLINE (Pubmed); (2) Scopus; (3) SPORTDiscus (EBSCO) and (4) Google Scholar. The main search string included the following words: (Spirulina OR Arthrospire OR Blue-Green Algae) AND (antioxidant OR antioxidants OR immunomodulatory OR immunomodulation OR immune system OR anti-inflammatory activities OR inflammation) AND (athlete OR athletes OR sport OR exercise OR physical exercise OR physical activity OR training). Moreover, the reference lists of included studies and pertinent reviews were also analyzed to identify further articles. The search was not restricted by date or publication status. Articles in English, Spanish, Italian and French were considered.

### Data collection

The results of the electronic search were evaluated independently by two reviewers (PC and GC). Duplicate studies were eliminated, and potentially eligible studies were identified and chosen based on title and abstract. Two reviewers (PC and GC) carefully screened the full text of the potentially eligible studies, and those that met the selection criteria were considered for the analysis. A third reviewer’s opinion was used to settle disagreements (VDO).

The following information was identified for each study by four reviewers (PC, GC, MD, and FL): author, year and country of publication, main purpose of the study, sample size, type of study design, participant characteristics (number, mean age, sex), supplementation dose, length of intervention and main results (Table 1).

**TABLE 1:** Sample and intervention characteristics of the studies.

Author, year, country	Study design	Sample characteristics (spirulina)	Sample characteristics (placebo and control group)	Daily dose, intervention length and placebo	Exercise protocol	Outcome	Main results
Chaouachi et al. (37), France	DB, parallel	*n* = 11, 25.8 ± 3.4 years, elite male Rugby Union players	*n* = 11, 26.3 ± 4.4 years, elite male Rugby Union players	5.7g, 7 weeks, 70.3% egg proteins and 29.7% carbohydrates	Concentric (three maximal knee extensions and flexions, at 60 and 240°s^–1^) and eccentric (three maximal contractions at 30°s^–1^) measurements with 2min rest between series, vertical jumps, running speed using a stationary 10-m and 30-m sprint test and Yoyo IRT-1.	Anthropometric measurements (BM, FM) and physical performance (isokinetic leg strength and power, VJ, speed and Yoyo IRT-1)	No significant differences between groups
Chaouachi et al. (20), France	DB, parallel	*n* = 9, 25.2 ± 2.7 years, elite male Rugby Union players	*n* = 8, 25.9 ± 3.3 years, elite male Rugby Union players	5.7g, 7 weeks, 70% egg proteins and 30% carbohydrates	Repeated high−intensity exercise bouts consisting of repeated 40 m (2 × 20 m) runs between markers set 20m apart, at a progressively increased speed.	Redox status (GSH, GSSG, SOD, GPx, ox-LDL and F2-Isop), inflammation (CRP and MPO) and muscle damage (CK and LDH)	↓CRP ↓CK ↓F2-Isop
Franca et al. (39), Brazil	DB, parallel	*n* = 11, 27.8 ± 3.5 years, male cyclists	*n* = 7, 34.3 ± 2.3 years, male cyclists	7.5g, 4 weeks, corn starch	High volume and intensity training regimes (six sessions/week, 2-6h per session)	Muscle damage (CK and LDH) and redox status (MDA and SOD)	No significant differences between groups
Gurney and Spendiff (41), England	DB, cross-over	*n* = 11, 21.1 ± 1.0 years, males untrained in arm cycling	*n* = 11, 21.1 ± 1.0 years, males untrained in arm cycling	6g, 7 days, soy protein	30-min bout of submaximal upper body cycling exercise at 55% V̇O_2*max*_, followed by an incremental test to fatigue using Arm Crank Ergometry.	Blood Hb, respiratory variables (RER, HR, Oxygen Uptake) and Time to fatigue	↑Hb ↑Oxygen Uptake ↓HR ↔ RER ↔Time to fatigue
Gurney et al. (42), England	DB, cross-over	*n* = 15, 40 ± 8 years, male cyclists	*n* = 15, 40 ± 8 years, male cyclists	6g, 21 days, microcrystalline cellulose	1-h submaximal endurance test at 55% external power output max and a 16.1km time trial (day 1), followed by a lactate threshold test and repeated sprint performance tests (day 2).	Blood parameters (Hb, glucose, lactate) respiratory variables (RER, HR, Oxygen Uptake) and exercise performance (time and power output)	↑Hb ↓HR ↑Power output (peak and average) ↔Oxygen Uptake ↔Time trial
Johnson et al. (40), USA	DB, parallel	*n* = 9, 20-43 years, active men	*n* = 8, 20-43 years, active men	3g, 8 weeks, gelatin capsules	30-min bout on a cross training, elliptical machine.	Physical (kcal in 30 min) and mental fatigue (15 min computer version of the Uchida–Kraepelin test).	↑ Physical fatigue (after 1 week) ↔ Physical fatigue (after 8 weeks) ↓Mental fatigue (both 1 and 8 weeks)
Juszkiewicz et al. (22), Poland	DB, parallel	*n* = 10, 20.4 ± 0.84 years, male Polish rowing team	*n* = 9, 20.0 ± 0.71 years, male Polish rowing team	1.5g, 6 weeks, calcium gluconate	2000-m time trial test on a rowing ergometer	Immune system (Tregs, CTLs, NK cells and Tδγ cells) and exercise performance (2000m time trial test)	↓Treg/CTL ↑Treg/(NK + Tδγ + CTL) ratio
Kalafati et al. (8), Greece	DB, cross-over	*n* = 9, 23.3 ± 1.7 years, male recreational runners	*n* = 9, 23.3 ± 1.7 years, male recreational runners	6g, 4 weeks, egg proteins	2h treadmill at 70%VO_2*max*_ + 95% VO_2*max*_ to exhaustion	Exercise performance (time to fatigue), exercise metabolism, redox status (GSH, GSSG, GSH/GSSG, TBARS, PC, CAT, TAC) and muscle damage (CK). (0, 1h, 24h, 48h post-exercise)	↓Carbohydrate oxidation rate ↑Fat oxidation rate ↑GSH (at rest and 24 h post-exercise) ↑Time to fatigue ↔CK
Kalpana et al. (38), India	DB, parallel	*n* = 30, 15-21 years, Indian male athletes	*n* = 30, 15-21 years control group (no supplementation), Indian male athletes *n* = 30, 15-21 years, group supplemented with commercially antioxidant (Selace Forte©, Indian male athletes	3g, 60 days, no placebo	Regular training mantained	Redox status (serum tocopherol, ascorbic acid, β-carotene, MDA).	↓MDA ↑Serum tocopherol ↑Ascorbic acid ↑β-carotene
Lu et al. (9), Taiwan	DB, parallel	*n* = 8, 20.00 ± 0.69 years, untrained students (3 male + 5 female)	*n* = 8, 21.43 ± 1.02 years, untrained students (3 male + 5 female)	7.5g, 3 weeks, soy protein	Exhaustive exercise (treadmill exercise following the Bruce incremental protocol)	Muscle damage (CK and LDH), redox status (MDA, GPx and SOD), blood lactate and time to exhaustion.	↑SOD ↓MDA ↑GPx ↑Time to exhaustion ↓LDH ↔CK
Pappas et al. (36), Greece	Parallel	*n* = 12, 22.5 ± 4.3 years, recreationally trained males	*n* = 12, 21.2 ± 2.2 years, recreationally trained males	6g, 4 days, wheat flour	Maximal eccentric voluntary contractions (5 × 15 with 2min rest) at an angular velocity of 60/s performed on an isokinetic dynamometer (knee range,0° full extension to 90° flexion)	Redox status (TAC and PC), muscle performance (EPT) and muscle damage (DOMS) (0,24h,48h,96h post exercise)	No significant differences between groups
Sadeghi et al. (43), Iran	Parallel	*n* = 12 (SP), 50.3 ± 2.9 years, inactive women *n* = 10 (SP + training), 51.5 ± 3.4 years, inactive women	*n* = 10 (control group, only training), 51.7 ± 2.3 years, inactive women	500mg, 12 weeks, no placebo	Regular training: 3d/week resistance training	Homocystein, anthropometry variables (waist to hip ratio and BMI)	↓Homocystein in SP + training compared to only SP or training
Sandhu et al. (24), India	DB, parallel	*n* = 10, 25.2 ± 3.5 years, untrained *n* = 10, 24.4 ± 3.4 years, trained n tot = 40 (22 men, 18 woman)	*n* = 10, 25.2 ± 3.5 years, untrained *n* = 10, 24.4 ± 3.4 years, trained n tot = 40 (22 men, 18 woman)	2g, 8 weeks, flour	2 isometric maximal voluntary contractions of the dominant quadriceps at 60° knee flexion with 10s and 60s hold with a rest period of 2 min.	Isometric strength (peak force and average force) and fatigue index.	↑Peak force ↑Average force ↔Fatigue index

BM, body mass; BMI, body mass index; CK, creatine kinase; CAT, catalase; CRP, C-reactive protein; CTL, cytotoxic lymphocytes; DB, double blind; DOMS, delayed onset muscle soreness; EPT, eccentric peak torque; F2-Isop, F2 α- isoprostanes; FM, fat mass; GPx, glutathione peroxidase; GSH, glutathione; GSSG, glutathione disulfide; h, hours; Hb, hemoglobin; HR, heart rate; IL-6, interleukin-6; IRT-1, intermittent Recovery Test Level 1; LDH, lactate dehydrogenase; MDA, malondialdehyde; min, minutes; MPO, myeloperoxidase; n, sample size; NK, natural killer; ox-LDL, oxidized low-density lipoprotein; PC, protein carbonyls; RER, respiratory exchange ratio; SOD, superoxide dismutase; TAC, total antioxidant capacity; TBARS, plasma thiobarbituric acid reactive substances; VJ, vertical jump; VO_2*max*_, maximal oxygen uptake.

### Quality assessment

Three authors (GC, PC, and MD) separately assessed the risk of bias of each included study using the revised Cochrane Risk-of-Bias tool for randomized trials (RoB2) (34). Disagreements were resolved by consultation with a third reviewer (FG) (34, 35).

## Results

### Study selection and characteristics

**FIGURE 1:**
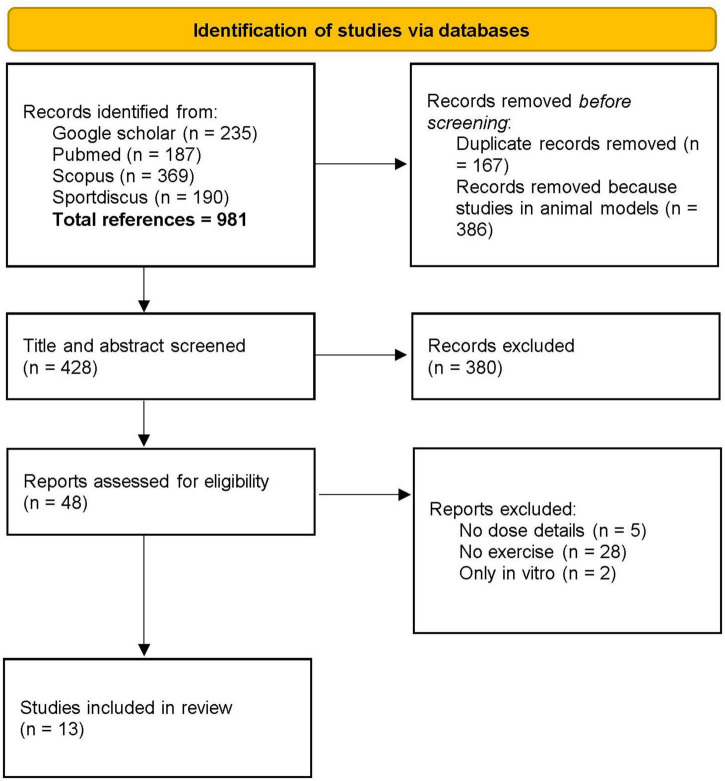
Prisma flow diagram of the selection of the articles included.

As shown in Figure 1, the primary search identified 981 relevant articles, 428 of which were assessed after duplicates had been removed and the titles and abstracts screened. According to the search topic and the inclusion criteria, 13 studies were included in the present systematic review (8, 9, 20, 22, 24, 36–43) (Table 1).

In total, 267 participants were analyzed. Only 3 studies involved female participants (9, 24, 43). The majority of the studies dealt with adult participants with a mean age of between 20 and 30 years; only two studies had participants with a mean age of 40 ± 8 (42) and 51 ± 3 years (43).

Spirulina (SP) supplements were mainly used at the dosages of 3 to 6 g/day, one study (9) used SP at higher dosages (7.5 g/day in college students) while three studies used less than 3 g/day of SP supplements (22, 24, 43). Most of the studies used SP supplements at the dosages of 1 to 6 g/day (ranging from 500 mg/d to 7.5 g/d). The duration of intervention ranged from 3 to 8 weeks in the majority of the studies (8, 9, 20, 22, 24, 37, 39, 40, 43), one study (43) was longer (12 weeks) and three studies lasted between 4 and 21 days (36, 41, 42).

Five studies involved athletes (20, 22, 37–39), five included trained subjects (8, 24, 36, 40, 42) and three were carried out on untrained people undergoing exercise (9, 41, 43).

**FIGURE 2:**
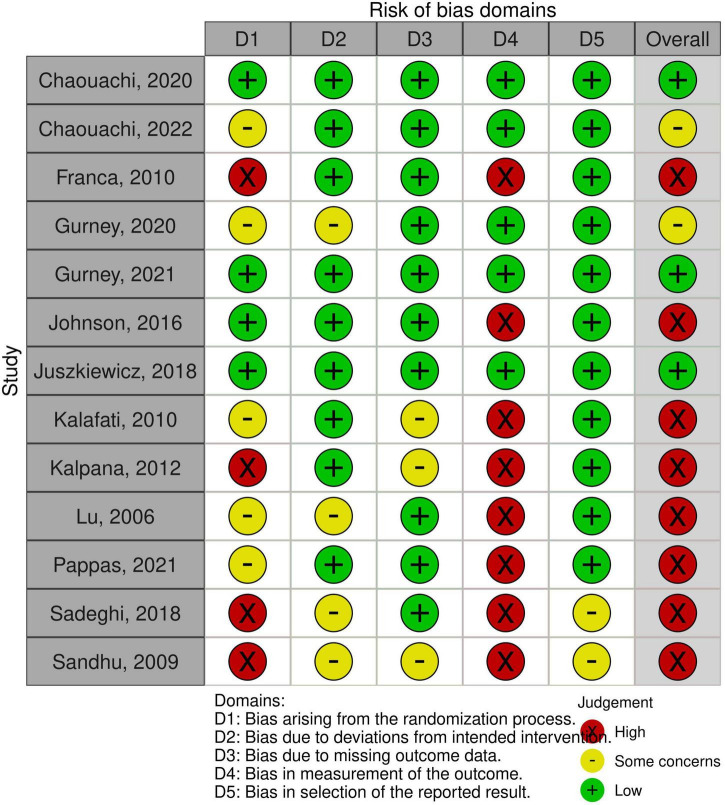
Methodological quality of the studies using the tool RoB 2.0.

As for the quality, three studies showed a low risk of bias, eight studies showed a high risk of bias and two studies had some concerns (Figure 2).

### Studies in athletes

Two studies, conducted by the same laboratory, reported the effects of SP supplementation in elite rugby players (20, 37). The first reported no significant difference between groups for squat jumps, countermovement jumps, and 10- and 30-m sprints after SP supplementation (37). The second demonstrated that SP supplementation prevents exercise-induced lipid peroxidation (F2-Isop), inflammation (CRP), and skeletal muscle damage (CK) and also accelerates the recovery of some of these markers immediately after and 24h after exhaustive exercise. However, other markers of redox state (SOD, GPx, oxidized low-density lipoprotein and glutathione/glutathione disulfide ratio or GSH/GSSG), inflammation (myeloperoxidase), and muscle damage (LDH), did not differ significantly between groups (20).

Two studies obtained opposing results, regarding protection against exercise-induced lipid peroxidation after SP supplementation (38, 39) in endurance exercise. In fact, SP did not interfere in the magnitude of oxidative stress (MDA and SOD) nor in muscle damage (CK and LDH) in regional-level cyclists, subjected to high volume and intensity of training (six sessions/week, 2-6h per session) (39).

However, the supplementation of SP resulted in a significant decrease in the level of MDA in young (aged 15–21) Indian male athletes (38). The same study demonstrated that supplementation of SP enhanced the levels of serum β-carotene, serum α-tocopherol, and plasma ascorbic acid in a similar way to a commercial antioxidant supplement (Selace Forte©, which mainly contained: Vit C-500 mg, Vitamin E-400 mg, Carotenoids-12.5 mg) (38).

Only one study, carried out on rowers of the Polish Rowing Team, showed that SP could influence the immune system (22). According to Juszkiewicz et al., SP may protect athletes against a deficit in immune function related to strenuous exercise by reducing the post-exercise increase in cells and regulatory T-cell count (22). In more detail, participants from the placebo group had a significant post-recovery increase in Treg/(NK cells + Tδγ + cytotoxic lymphocytes) ratio, which was absent in the SP group. Nonetheless, no ergogenic effect was observed in a 2000-m rowing ergometer test (22).

### Studies in trained subjects

Two studies explored the effect of SP supplementation on muscle strength, reporting different results (24, 36). According to Sandhu et al., SP was effective in increasing peak isometric muscle strength, average force and reducing fatigue similarly in trained and untrained volunteers (24). On the other hand, in recreationally trained males, SP did not confer beneficial effects on peak torque (EPT), redox status [Total antioxidant capacity (TAC) and PC] or delayed onset muscle soreness (DOMS) immediately after exercise, as well as at 24, 48, 72, and 96 h post exercise (eccentric contractions consisting of 5 sets and 15 maximum reps per set) (36). Some authors observed an improved exercise tolerance [time to fatigue at 95% of maximal oxygen uptake (VO_2_max) after a 2-h run at 70%–75% of VO_2_max] in recreational runners after SP supplementation together with an attenuated exercise-induced increase in lipid peroxidation (TBARS) (8). However, they failed to show any significant effect on muscle damage (CK) after the exercise protocol (8).

In trained cyclists, SP significantly increased hemoglobin (Hb) and reduced heart rate (HR) during submaximal exercise (42). Although results did not show improvement in exercise performance (16.1km time trial after 1-h submaximal endurance test), SP supplementation significantly increased power output during repeated sprint performance tests (42).

One study reported an improvement in indices of mental fatigue in men 4 h after the first supplementation as well as 8 weeks later and also a statistically significant increase in exercise output (Kcals consumed in 30 min exercise on a cross trainer machine) after SP supplementation (40).

### Studies in untrained people

Sadeghi et al. found that improvement in homocysteine levels were significantly greater in inactive females after SP combined training (43) compared with only training (43). The training protocol included three sessions a week of supervised training consisting of resistance exercise plus steady-state exercise performed at an intensity of 60–70% maximal heart rate for 30 min.

One study examined the effects of SP on redox status and muscle damage in college students by comparing the results of the Bruce incremental treadmill exercise before and after SP treatment (9). The results showed that plasma concentrations of MDA and LDH were significantly lower after supplementation with SP, while the activity of SOD and GPx significantly improved. Furthermore, time to exhaustion (TE) was significantly extended, leading the authors to speculate that ingestion of SP may confer protection against muscle damage related to exercise (9).

Spirulina (SP) supplementation significantly enhanced oxygen uptake and Hb during arm cycling submaximal exercise (30-min submaximal exercise bouts, corresponding to 55% of V̇O_2_max, followed by an incremental test to fatigue) in males untrained in arm cycling (41). However, time taken to fatigue was not different.

## Discussion

The studies analyzed by this review have been written in the last 20 years and evaluate the exercise-related effects of SP supplementation. Antioxidant, anti-inflammatory, immunomodulatory and ergogenic effects of SP have been observed. Research findings on the use of SP in sports, with a focus on ergogenic effects and influence on recovery and the immune system, are summarized in Table 2.

**TABLE 2:** Summary of research findings on the use of Spirulina in sport.

Ergogenic effect	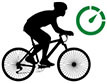	A supplementation of 6–7.5 g/day could be considered in athletes engaged in a high volume of submaximal endurance training (e.g., cyclists or runners) in order to improve redox status, fatigue tolerance and hemoglobin level (8, 9, 41, 42). SP supplementation does not seem to improve physical performance in power athletes (22, 37).

Recovery	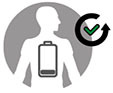	The majority of studies showed no benefit on CK (8, 9, 39) or DOMS (36) suggesting no implication in muscle recovery. 5.7g/day of SP seems to accelerate recovery after training/competitions in elite rugby players (20). SP supplementation may potentially prevent exercise-induced oxidative damage, inflammation, and muscle damage in elite athletes who do not achieve the recommended antioxidant dietary intake (20, 38).

Immune system	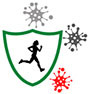	It is hypothesized that SP can protect athletes against immune dysfunction associated with heavy exercise. SP may play a role in the maintenance of lower Treg counts in tissues, preventing the immunosuppressive effects of these cells and restoring an immune balance (22). Currently, evidence supporting the benefit to the immune system through SP supplementation remains scarce.

CK, creatine kinase, DOMS, delayed onset muscle soreness, SP, spirulina.

### Antioxidant effects

Evidence on the antioxidant effects of SP is mixed. According to Kalafati et al., SP supplementation improved GSH concentration and attenuated the exercise-induced increase in lipid peroxidation, thereby inducing a significant improvement in exercise performance (8). However, authors reported a lower daily intake level of vitamin A and selenium in the SP group. In concert with vitamin E and GPx, selenium helps minimize the generation of harmful free radicals, especially during endurance exercise, hence benefits in the SP group may have been influenced by differences in daily antioxidant dietary intake between groups (placebo and SP) (8). Other findings suggested that SP supplementation did not confer beneficial effects on redox status, performance, or muscle damage following a muscle damaging protocol based on eccentric exercise (36). However, it should be considered that a single bout of exercise based on only eccentric contraction does not represent real life conditions.

Two studies agreed that SP supplementation reduces oxidative damage by significantly lowering MDA concentrations and raising blood SOD activity (9, 38). The supplementation of SP also enhanced the levels of antioxidants in the blood with greater benefits on the redox state in endurance than in mixed sports (38). In this case, it is important to note that all participants’ intake of β-carotene, iron, and zinc was lower than the recommended daily allowance (38). Indeed, similar benefits on SOD and MDA were not observed in cyclists with adequate nutritional status (39) or elite rugby players (20).

Overall, there are several things to point out. First, many studies used TBARS, TAC or MDA to assess lipid peroxidation (8, 9, 36, 38, 39). Lipid peroxidation is still a valuable marker of exercise-induced macromolecule damage (44), however, considering that most MDA is generated by the assay itself (i.e., artificial lipid peroxidation), two or more indices are usually required to confirm lipid damage (45). Furthermore, the TBARS and TAC assays are no longer recommended for assessing lipid peroxidation (46) and should be discontinued as strongly advised by many experts (31, 46). In addition, elevated levels of GSH/GSSG, which are often used as a marker of oxidative stress after exercise, have also been reported due to methodological errors (47).

Equivocal results could potentially be related to inter-individual baseline variability in antioxidant compounds prior to beginning supplementation. In this sense, results should be carefully interpreted based on participant characteristics, protocol used and control group or placebo.

Lastly, supplementation for redox balance management during and after exercise is controversial since it may interfere with numerous ROS-mediated mechanisms that influence training adaption, including mitochondrial biogenesis and hypertrophy (11, 48–52). Accumulating evidence suggests that exercise-induced reactive species are required for the activation of signals regulated by redox-sensitive transcription factors (PCG-1, HIF-1α, NF-κB, and NFE2L2) (13, 50, 53). There has been no research into how SP may interfere with ROS-mediated adaptations such as strength, hypertrophy, or endurance, and further investigation is urgently needed in this sense.

Although it is clear that SP has a wide range of components capable of free radical scavenging *in vitro*, there is still a significant knowledge gap regarding the antioxidant benefits of SP supplementation in athletes or people engaged in exercise in general (31).

### Effects on inflammation

Beyond redox state, four studies explored the anti-inflammatory action of SP (8, 9, 20, 39). Of these studies, two involved athletes (20, 39). Indeed, an inflammatory response (54) associated with muscle damage during and after exercise exists, which leads to increases in inflammatory markers like CRP (55–57) and intracellular proteins like CK (55), and the release of cytokines like tumor necrosis factor (TNF-α) and IL-6 in order to repair damaged tissue (58). This inflammatory status frequently causes muscle pain and functional decline (55), impairing overall performance (51, 59).

Studies in animal models have reported an anti-inflammatory effect of SP (60, 61) comparable to that of diclofenac sodium (60). In humans, results from a recent meta-analysis of controlled clinical trials demonstrated that SP supplementation resulted in a significant reduction of IL-6 concentrations when the baseline body mass index (BMI) of participants was lower than 25 kg/m^2^ (62). Almost none of the studies included in our work that evaluated the effects of SP on muscle damage showed any benefit on CK (8, 9, 39) and DOMS (36), suggesting no implication in muscle recovery. Only recently, SP supplementation has been shown to have a role in the prevention of exercise-induced inflammation and skeletal muscle damage by reducing CK, CRP and F2-Isop levels immediately and 24h after exercise in elite rugby players (20). Although these results seem to be favorable, the mechanisms behind the above-mentioned effects of SP related to exercise are still poorly understood. According to our systematic review, no studies have yet considered the effects of SP on TNF-α, IL-6, and CRP in healthy subjects and/or athletes, and more research should be conducted in this area.

### Immunomodulatory properties

Innate and acquired immunity have both been reported to decline temporarily in the hours following strenuous exercise (by up to 70%) basically giving an “open window” for opportunistic infections (29). Effector lymphocytes, such as NK cells, T lymphocytes (helper and cytotoxic), TCR δγ-positive (Tδγ) cells and regulatory T cells (Tregs) play a crucial role in cell-mediated immune response (29). Some authors have documented SP’s ability to affect the immune system in healthy individuals (23, 63). In 2009, Milasius et al. reported for the first time that SP supplementation had a positive effect on the quantitative indices of immune response in high performance sportsmen (21).

Exposure to a high stressor, such as maximal exercise, nonetheless results in an increase/decrease both in the number of cytotoxic cells and in Treg count. Based on the results of Juszkiewicz et al., SP supplementation did not exert a statistically significant effect on Treg count in members of the Polish Rowing Team (22). However, athletes in the placebo group showed a significant post-recovery increase in the Treg/(NK + Tδγ + cytotoxic) ratio, which was lacking in the supplemented group, leading the authors to speculate that SP supplementation may protect athletes from immunological deficiencies. Currently, the study published by Juszkiewicz and colleagues remains the only randomized study to have investigated the effects of SP in athletes (22).

There is little evidence to support the idea that athletes should suppress their immune systems, yet taking supplements to boost immune systems is still a controversial topic due to the multiple mechanisms that modulate immune response during and after exercise (29). According to a new paradigm for exercise immunology proposed by Walsh that considers “resistance” (the strength of the immune weaponry) and “tolerance” (the ability to endure microbes and dampen defense activity), it is not surprising that supplements designed to increase immune “resistance” have little or no efficacy (29). There is growing knowledge that episodes of upper respiratory symptoms (URS) usually cluster around intense periods of training or competition (64–66), especially during winter months (66, 67) leading to respiratory inflammation, characterized by a dysregulated anti-inflammatory response and oxidative stress (68). Although athletes generally consume a nutrient-dense diet rich in fruit and vegetables (69, 70), SP may improve tolerance, mitigating tissue damage during exercise or infection, and improve recovery (67). New studies should be designed to explore the potential tolerogenic properties of SP, focusing on the prevention or treatment of URS.

The efficacy of immunonutrition techniques should be investigated using multi-omics approaches. As pointed out by many authors, metabolomics, lipidomics, and proteomics allow the simultaneous evaluation of a large number of small-molecule metabolites, lipids, and proteins, providing a system-wide overview of the metabolic response to exercise and nutritional interventions (71–73). Future studies should contemplate the use of a human systems biology approach with multi-omics outcomes to better understand whether or not SP can aid athletes.

Nowadays, evidence supporting the benefit of SP supplementation on the immune system remains scarce and it is still too early to encourage its use for this purpose.

### Ergogenic effect

Although antioxidant benefits are typically the main focus of SP supplementation, several authors have reported promising results of SP as an ergogenic aid, proposing multiple pathways that may be responsible for the observed improvements. For instance, there is growing evidence that SP can improve submaximal exercise and fatigue tolerance (8, 9, 40, 42). These benefits could be attributable to several factors. Firstly, an ergogenic effect may be due to the antioxidant effect of SP: the contraction-induced ROS generation is associated with oxidative damage and earlier onset of fatigue (11, 15). Unsurprisingly, improvements in exercise performance were often observed together with an enhanced redox state at rest after SP supplementation (8, 9). Some evidence in rats has shown that SP can have vasodilatory effects. For instance, SP could enhance circulatory nitrate/nitrite, increasing nitric oxide availability and the expression of endothelial nitric oxide synthase (74, 75). However, despite enhanced nitrate intake potentially representing a good strategy for improving performance (76–79), increases in nitrate/nitrite following SP supplementation in humans have not yet been reported.

Secondly, the high iron concentration of SP may contribute to its ergogenic effects. It is commonly recognized that adequate iron stores are critical for regular Hb synthesis and that Hb is essential for the transport of oxygen from the lungs to the muscles (80). Due to the lack of phytates and oxalates, SP iron is easily absorbed by the body. This has led to some positive hemopoietic trends in athletes and healthy, active men without a known iron deficiency (21, 41). In particular, some authors have demonstrated that SP supplementation may enhance oxidative capacity (8) and significantly increase Hb during submaximal exercise (41, 42). To the best of our knowledge, SP has exclusively been investigated in male athletes and, considering the high prevalence of anemia in female athletes, future studies should involve female participants, as recently highlighted by Gurney and Spendiff (31).

Finally, SP supplementation could improve performance by alleviating mental fatigue, as shown by Johnson et al. (40). However, the evaluation of fatigue remains a very complex topic. As recently proposed, fatigue should be defined in terms of fatigability (perceived and performance fatigability) and should not be combined with any adjective, such as mental fatigue, muscle fatigue or physical fatigue (81, 82). Further studies designed with protocols that allow the assessment of performance fatigability during exercise are encouraged (83–85), in order to understand the anti-fatigue implications of SP supplementation.

Considering the effects of SP on muscle strength and power performance, all studies but one (24) failed to show significant improvements (22, 36, 37). SP supplementation was effective in increasing peak force and average force of the quadriceps in trained and untrained individuals. Nevertheless, the psychological and motivational components of the subject during the testing of maximal voluntary isometric contraction might have affected the outcome of the study, as reported by the authors in the limitations of their study (24). In elite male rugby players during the competitive phase, SP supplementation did not improve maximal strength and power (37). Also, in members of the Polish rowing team, SP supplementation did not show ergogenic effects (22). Differences in doses (1.5g–6g), intervention lengths (4 days–8 weeks), training status (from untrained to elite athletes) and protocols, make it difficult to compare findings from studies evaluating the impact of SP on muscle strength and power performance.

## Strengths and limitations

This systematic review included all published research that has looked at the effects of SP supplementation considering its antioxidant, immunomodulatory, and anti-inflammatory activities, underlying biochemical mechanisms and practical implications. The reviews on this topic often focused on a single mechanism of action of SP cataloging its effects only at the level of some biological compounds or on some clinical aspects. With this systematic review we wanted to focus on the proven main properties of this compound analyzing the known effects of SP on immune response and inflammatory processes in athletes, and on the consequences that it may have in terms of athletic performance and recovery.

However, when interpreting our findings, several limitations should be acknowledged. First, given the specificity of the target – athletes and people doing exercise, we decided to analyze the literature regarding the anti-oxidant, immunomodulatory and anti-inflammatory effects of SP in these groups separately from the other records found following the registered protocol. As a consequence, this systematic review included 13 studies with only 267 study participants, and the majority of the trials involved small groups of subjects randomly allocated to parallel groups. Furthermore, in the included articles, SP was given in varied quantities or forms for a variable duration of intervention and even the characteristics of study participants varied widely. Moreover, the different exercise protocols and participants’ training status make it difficult to compare results. Finally, it is important to underline that the quality of these studies was generally poor; indeed, only three studies showed a low risk of bias. All these aspects hinder the validity of our findings and do not allow to express definite evidence on the use of SP in exercise and sport. New, higher quality research is needed to understand under what conditions and in what type of individuals SP supplementation may be recommended.

## Conclusion

Considering the effects of SP on exercise-related oxidative stress, equivocal results could potentially be related to inter-individual baseline variability in antioxidant compounds, participants’ characteristics (e.g., age and sex), and training status. Based on this, there is still a significant knowledge gap regarding the antioxidant benefits of SP supplementation in athletes or people engaged in exercise in general.

Emerging evidence suggests that SP could be useful during submaximal endurance exercise, increasing oxygen uptake and improving exercise tolerance; on the other hand, SP supplementation does not seem to enhance physical performance in power athletes.

As for the anti-inflammatory and immunomodulatory effects, the majority of the studies suggest the lack of SP benefits on muscle recovery, and despite the idea that SP supplementation may protect athletes from immune dysfunction associated with intense exercise, evidence supporting benefits on the immune system from SP supplementation is still lacking.

Therefore, evidence for promoting SP consumption in healthy subjects to improve athletic performance and accelerate recovery is still scarce; thus, at this stage, it could only be suggested in elite athletes who do not achieve the recommended antioxidant dietary intake to improve deficiencies and/or nutritional status.

### Future directions

In order to better explore the impact of SP on healthy subjects involved in exercise, we recommend that research:

1. Use multiple biomarkers to assess oxidative stress or redox signaling and abandon outdated assays (i.e., TBARS and TAC).
2. Evaluate interference with training adaptation (e.g., hypertrophy, strength and mitochondrial biogenesis).
3. Assess benefit on muscle damage, and recovery in real life or similar conditions (e.g., during a competition, high-intensity training period or consecutive simulated games).
4. Evaluate impact on prevention or treatment of URS, especially in winter.
5. Contemplate the use of a human systems biology approach with multi-omics outcomes to have a system-wide overview of the adaptive response to exercise and nutritional interventions.
6. Explore the ergogenic aids in female athletes.

## Data availability statement

The original contributions presented in this study are included in the article, further inquiries can be directed to the corresponding author.

## Author contributions

PC, VDO, and FG: conceptualization. PC, GC, and FG: methodology. PC, MD, GC, and FL: formal analysis. PC, GC, and MD: data curation. PC and GC: writing—original draft preparation. PC, GC, MD, FL, VDO, FG, and GL: writing—review and editing. VDO, FG, and GL: supervision. GL: funding acquisition. All authors read and agreed to the published version of the manuscript.
